# The complete chloroplast genome sequence of *Actinidia arguta* var*. giraldii*

**DOI:** 10.1080/23802359.2020.1870884

**Published:** 2021-02-09

**Authors:** Fangbing Ding, Lei Zhang, Yaling Wang, Yongpeng Wu, Fengwei Wang, Yu Zhao, Ying Zhang

**Affiliations:** a Xi’an Botanical Garden of Shaanxi Province, Institute of Botany of Shaanxi Province, Xi’an, PR China; b Shaanxi Engineering Research Centre for Conservation and Utilization of Botanical Resources, Xi’an, PR China

**Keywords:** *Actinidia arguta* var. giraldii, chloroplast genome, phylogenetic analysis, Actinidiaceae

## Abstract

The complete chloroplast (cp) genome sequence of *Actinidia arguta* var*. giraldii* (Diels) Vorosch. was assembled and characterized by Illumina pair-end sequencing date. The complete plastid genome was 156,729 bp in length, containing a large single-copy (LSC) of 89,647 bp, and a small single-copy (SSC) of 22,482 bp, which was separated by a pair of 22,300 bp inverted repeat regions (IR). A total of 131 unique genes were identified, including 84 protein-coding genes, 39 tRNA genes, and 8 rRNA genes. The phylogenetic position based on the chloroplast genome of 11 species showed that *A. arguta* var*. giraldii* was sister to *Actinidia kolomikta*.

*Actinidia arguta* is a climbing, perennial and dioecious vine, native from northeast Asian countries(China, Japan, Korea, and Siberia) (Zhu et al. 2019) and has become increasingly popular due to its exceptional flavor, interesting phytochemical profile, and outstanding pro-healthy properties (Latocha et al. 2015; Wojdyło and Nowicka 2019). Hardy kiwi is one of the most nutritionally rich fruits and an excellent source of antioxidants (mainly polyphenols), vitamins (especially vitamin C), carotenoids, chlorophylls, sugars, dietary fiber, organic acids, and minerals (Leontowicz et al. 2016; Latocha 2017; Diana et al. 2020). Hardy kiwi has been associated with the preventive effects of various chronic diseases, such as cardiovascular and digestive disorders. (Latocha et al. 2015; Latocha 2017). *A. arguta* var. *giraldii* (Diels) Vorosch. is a variety of *A. arguta*, mainly distributed in Shaanxi province in China, growing at an altitude of 1000 m. It produces small grape-sized fruits with thin, edible, and hairless skin and aromatic sweet flavor. These fruits come in green, yellow, or purple as it ripens which provides valuable materials to study the color of kiwi. The exploration of complete chloroplast genomes of it can provide a methodological guidance for evolution and phylogenetic analysis of *Actinidia* in the future.

The materials were introduced from Heilong River, Guyi District, Xi’an City, Shaanxi Province (108°46′E, 33°87′N) and the voucher specimens were stored at Xi’an Botanical Herbarium (XBGH). Total genomic DNA was extracted from young leaves using the modified CTAB method (Doyle 1987). Genome sequencing was performed using Illumina HiSeq 4000 platform at Biomarker Technologies Corporation (Beijing, China). The qualified cleaned reads were assembled with NOVOPlasty (Dierckxsens et al. 2017) and modified using Geneious Prime version 2020.2 (https://www.geneious.com). Then the annotation was performed by Plann (Huang and Cronk 2015) and Geneious Prime version 2020.2 based on the annotation of *A. arguta* (NC_034913.1). Finally, the validated complete chloroplast genome of *A. arguta* var*. giraldii* was deposited in Genbank (accession number MT890912).

The complete chloroplast genome of *A. arguta* var*. giraldii* is 156,729 bp in length, including a large single-copy region (LSC) of 89,647 bp and a small single-copy region (SSC) of 22,482 bp, and two 22,300 bp inverted repeat regions (IR). A total of 131 genes are annotated, containing 84 protein-coding genes, 39 tRNA genes, and 8 ribosomal RNA genes. The overall GC content of *A. arguta* var*. giraldii* chloroplast genome is 50.1%.

To confirm the phylogenetic position of *A. arguta* var*. giraldii*, ten chloroplast genome sequences of Actinidiaceae and one sequence of *Saurauia* were aligned by MEGA version 7.0 (Kumar et al. 2016). The result indicated that *A. arguta* var*. giraldii* was found to be relatively closely related to *Actinidia kolomikta* compared to other species of *Actinidia* genera in Actinidiaceae (Figure 1). Our results defines the phylogenetic position of *A. arguta* var*. giraldii* at the molecular level, further improves the chloroplast genome information in *Actinidia* and provides fundamental information for further phylogenetic researches and exploitation, utilization of *Actinidia*.

**Figure 1.**
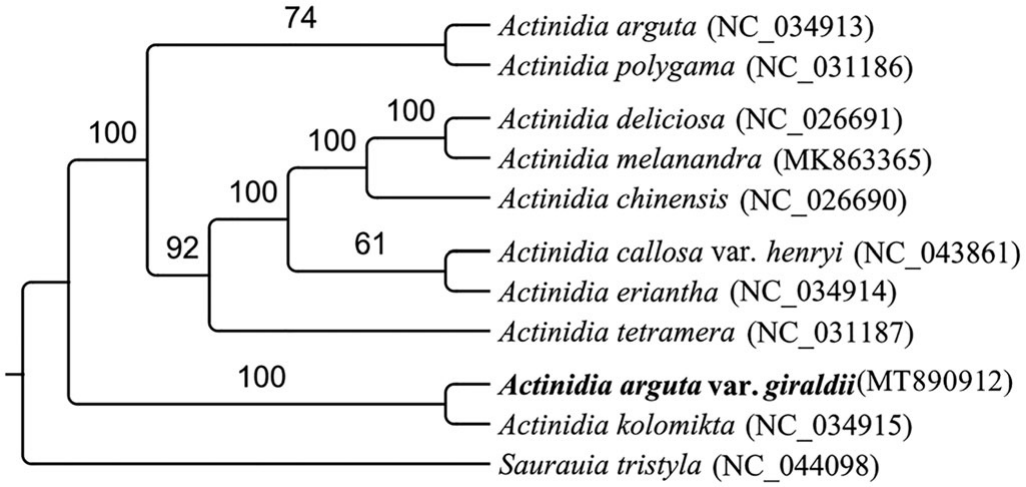
The phylogenetic tree based on the complete chloroplast genome sequences of 11 species. The *Actinidia arguta* var. g*iraldii* is marked in bold type and bootstrap support values are listed above each branch.

## Data Availability

The genome sequence data that support the findings of this study are openly available in GenBank of NCBI at (https://www.ncbi.nlm.nih.gov/) under the accession no. MT890912. The associated BioProject, SRA, and Bio-Sample numbers are PRJNA677853, SRR13039560, and SAMN16745906, respectively.
